# Detection and Analysis of RNA Ribose 2′-*O*-Methylations: Challenges and Solutions

**DOI:** 10.3390/genes9120642

**Published:** 2018-12-18

**Authors:** Yuri Motorin, Virginie Marchand

**Affiliations:** 1 UMR7365 IMoPA, Biopôle, CNRS-Lorraine University, 9 Avenue de la Forêt de Haye, 54505 Vandoeuvre-les-Nancy, France; 2 UMS2008 IBSLor, Biopôle, CNRS-Lorraine University-INSERM, 9 Avenue de la Forêt de Haye, 54505 Vandoeuvre-les-Nancy, France; virginie.marchand@univ-lorraine.fr

**Keywords:** RNA 2′-*O*-methylation, ribose methylation, detection, deep sequencing, quantification, RiboMethSeq, Nm-Seq, RibOxi-Seq, 2′OMe-Seq

## Abstract

Ribose 2′-*O*-methylation is certainly one of the most common RNA modifications found in almost any type of cellular RNA. It decorates transfer RNAs (tRNAs), ribosomal RNAs (rRNAs), small nuclear RNAs (snRNAs) (and most probably small nucleolar RNAs, snoRNAs), as well as regulatory RNAs like microRNAs (miRNAs) and Piwi-interacting RNAs (piRNAs), and finally, eukaryotic messenger RNAs (mRNAs). Due to this exceptional widespread of RNA 2′-*O*-methylation, considerable efforts were made in order to precisely map these numerous modifications. Extensive studies of RNA 2′-*O*-methylation were also stimulated by the discovery of C/D-box snoRNA-guided machinery, which insures site-specific modification of hundreds 2′-*O*-methylated residues in archaeal and eukaryotic rRNAs and some other RNAs. In this brief review we discussed both traditional approaches of RNA biochemistry and also modern deep sequencing-based methods, used for detection/mapping and quantification of RNA 2′-*O*-methylations.

## 1. Introduction

2′-*O*-methylation is a highly common modification in different cellular RNAs, present in transfer RNAs (tRNAs), ribosomal RNAs (rRNAs), small nuclear/small nucleolar RNAs (snRNAs/snoRNAs), as well as in microRNAs (miRNAs)/Piwi-interacting RNAs (piRNAs) and some messenger RNAs (mRNAs) (for review [1]). The enzymatic machinery implicated in 2′-*O*-methylation (2′-*O*-Me) is rather complex and diversified. RNA methylation is insured both by stand-alone protein enzymes and small nucleolar ribonucleoprotein (sno(s)RNP) complexes integrating a C/D-box sno(s)RNA guide and a common catalytic subunit (Nop1/fibrillarin). These aspects of RNA methylation as well as numerous biological functions of 2′-*O*-methylation are discussed in details elsewhere [1].

**Figure 1:**
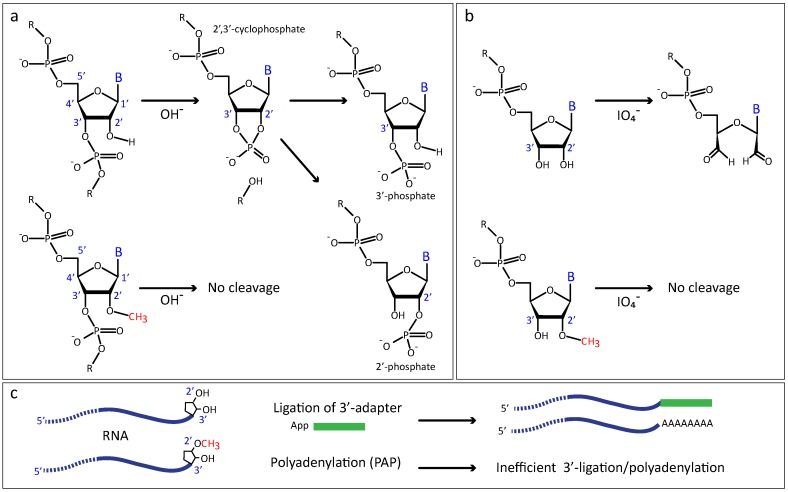
Chemical and enzymatic properties of 2′-*O*-methylated RNA chains (**a**) Internal 2′-*O*-methylation increases the resistance of RNA to nucleolytic cleavage at alkaline conditions; (**b**) resistance of 3′-terminal 2′-*O*-methylated residues to periodate (IO_4_^−^) oxidation; (**c**) RNA ligase and polyA-polymerase activities are affected at the 2′-*O*-methylated RNA 3′-terminal residues.

Ribose 2′-*O*-methylation (RNA 2′-*O*-methylation, Figure 1a,b), confers to the RNA polynucleotide chain particular physico-chemical properties and specific reactivity, which differ considerably from unmodified RNA. Most of these changes are now exploited for specific detection of ribose 2′-*O*-methylation.

First of all, the presence of a methyl (-CH_3_) group at the 2′-OH of the ribose preferentially stabilizes 3′-endo ribose conformation, typical for nucleotides in A-type RNA chain [2,3,4,5,6]. Secondly, methylation of the ribose 2′-OH almost completely abolishes the nucleophilic property of the 2′-OH oxygen atom, leading to a greatly increased resistance of the 3′-adjacent phosphodiester bond to alkaline hydrolysis (Figure 1a), as well as to nuclease cleavage (RNase T_2_ and RNase H, for example). Thirdly, when a 2′-*O*-Me is present at the 3′-terminal nucleotide, the methyl group prevents the coordination with bidentate oxidative agents, such as periodate (IO_4_^−^) ion, and thus protects the terminal ribose [7] (Figure 1b). Such 3′-terminal 2′-*O*-methylation also negatively affects the ligation efficiency by RNA ligase [8,9] and the 3′-end activity of polyA-polymerase [10,11] (Figure 1c).

Even if 2′-*O*-Me is not directly affecting base-pairing properties of nucleotides, minor steric hindrance at the ribose moiety becomes important during primer extension, especially at low deoxynucleotide triphosphates (dNTP) concentration. Thus, under these specific conditions, many natural RNA-dependent DNA polymerases (reverse transcriptases, RT-enzymes) show sensitivity to 2′-*O*-methylation in the RNA template [12].

## 2. Classical Methods for 2′-*O*-Methylation Detection

The presence and stoichiometry of 2′-*O*-methylated nucleotides in RNA can be detected and quantified both by general analytical approaches of RNA biochemistry, and by specific applications exploiting particular chemical properties of 2′-*O*-methylation.

### 2.1. General Methods of RNA Analytical Chemistry

The presence of 2′-*O*-methylated nucleotides in RNAs was initially detected by different approaches, mostly based on general analysis of nucleotide(-side) composition of cellular RNAs. For example, total RNA hydrolysis by perchloric acid (HClO_4_) followed by a measurement of released methanol, was proposed for specific quantification of RNA 2′-*O*-methylation [13]. Other specific methods from this period used partial hydrolysis followed by oligonucleotide analysis coupled with periodate oxidation [14].

Similar to all other modified nucleotides in RNAs, 2′-*O*-Me can be also detected by various types of chromatography [15,16] as well as by techniques of mass-spectrometry [17,18,19,20], which are not particularly specific for those modified residues, but still allow their detection and, in some instances, quantification (reviewed in [21,22,23]).

From 70–80′s, two-dimensional thin layer chromatography (2D TLC) was massively employed for separation of mononucleotide 5′-phosphates obtained by enzymatic hydrolysis of RNAs and radioactively labeled in vivo (pre-labeling) or in vitro (post-labeling) (for examples see [24,25,26]). It is noteworthy, that 5′-Nm-N-3′ link is stable upon RNase T_2_ hydrolysis and thus, when RNase T_2_ is used, labeled di-nucleotides containing 2′-*O*-methylation have to be analyzed instead of mononucleotide 3′-phosphates. For the analysis of long RNAs, RNA fingerprinting analysis was frequently employed [27,28,29].

Recent examples of analytical strategies include isolation and analysis by combination of high-performance liquid chromatography with mass spectrometry (HPLC/MS) of mung bean or RNase T_1_ fragments of rRNA, an approach which allowed to finalize the modification profile of yeast *Saccharomyces cerevisiae* and human ribosome [30,31,32,33]. Even if the analyses were done using different cell lines and certainly different conditions of culture, modern mass-spectrometry approaches [32,33] demonstrate an excellent correlation with the quantitative methylation data obtained by various RiboMethSeq protocols (R^2^ > 0.98, see Supplementary Figure S1).

### 2.2. Specific Detection Strategies

Specific detection strategies are mostly based on particular chemical properties of 2′-*O*-methylated nucleotides listed above.

#### 2.2.1. Increased Resistance to Alkaline or Enzymatic Hydrolysis

**Figure 2:**
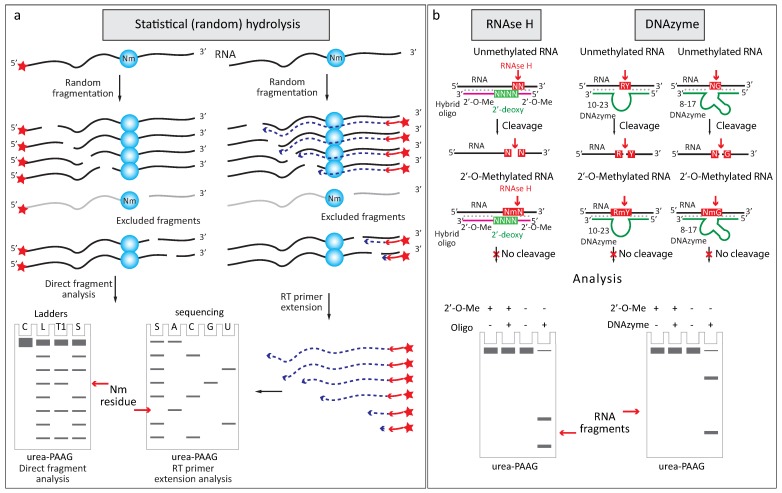
Analysis of 2′-*O*-methylation using resistance to nucleolytic cleavage (**a**) Random partial RNA hydrolysis generates “gaps” in the RNA ladder, an analysis can be done by direct RNA labeling (left) or by primer extension using a labeled DNA primer (right). To locate the exact position in the sequence, ladder with fragmented unmodified RNA (L) and RNase T1 cleavage (T1) was used in the case of directly labeled RNA (left). For primer extension, RNA sequencing with the same primer was used (right); (**b**) Guided cleavage by RNase H or DNAzyme is compromised when the nucleotide in RNA is 2′-*O*-methylated. Cleavage by RNase H is directed by hybrid synthetic oligonucleotide composed of 2′-*O*-Me residues and internal ‘window’ of four standard DNA nucleotides (indicated in green). Depending on the source of RNase H and the exact experimental conditions, the exact cleavage position in the duplex may vary [40]; the most common site is shown on Figure 2. DNAzymes 10–23 and 8–17 are standard DNA oligonucleotides, 10–23 version cleaves Purine-Pyrimidine (RY) sequence context, 8–17 variant acts at any nucleotide-G (NG) contexts. Nm: 2’-*O*-methylated nucleotide.

As mentioned above, this particular property was already extensively used in the past to isolate alkali- (or nuclease-) stable 5′-Nm-N-3′ dinucleotides for global RNA modification analysis [34,35]. Later, it was noticed that when a modified RNA is subjected to a random (statistical) alkaline hydrolysis, 2′-*O*-Me groups generate characteristic “gaps” in the random cleavage profile, due to the relative protection of 3′-adjacent phosphodiester bond [12,36,37,38,39]. An analysis of such cleavage profiles can be performed either by using purified and end-labeled RNA, or directly on total RNA fraction, using specific primer-extension with RT-enzyme (Figure 2a).

Resistance of 2′-*O*-methylated RNA to alkaline cleavage was later exploited in site-specific detection approach based on the use of RNase H [40,41] and two DNA-based ribozyme (DNAzyme) versions for specific RNA cleavage [42] (Figure 2b).

#### 2.2.2. Reverse Transciptase Stop at Low Deoxynucleotide Triphosphates Concentration

**Figure 3:**
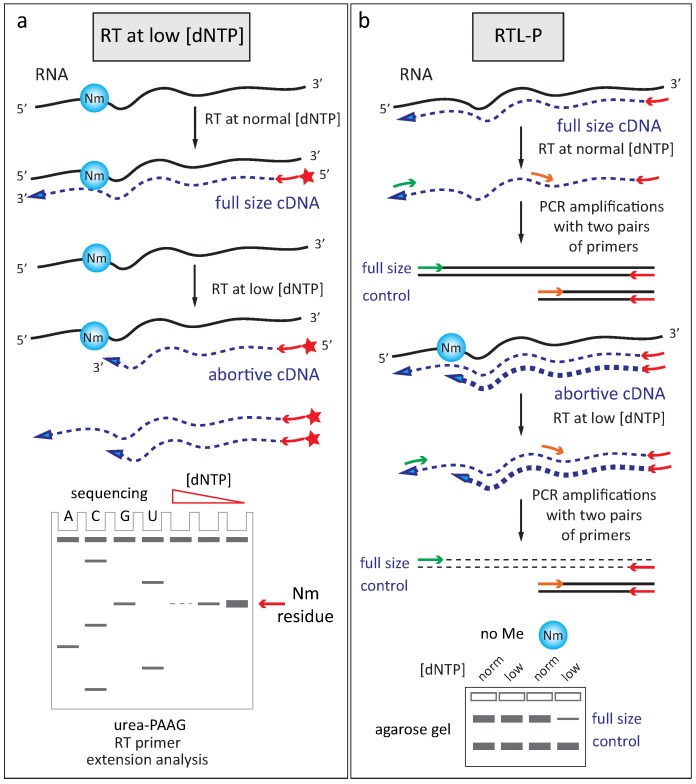
Analysis of 2′-*O*-methylation by reverse transcriptase (RT) stop at low [deoxynucleotide triphosphate (dNTP)]. (**a**) RT pauses at a 2′-*O*-methylated residue at low [dNTP], analysis of abortive complementary DNA (cDNA) chains is done by urea-PAAG; (**b**) Semi-quantitative PCR amplification of full-length and abortive cDNA chains obtained at normal and low [dNTP].

With the development of RT-dependent primer extension on RNAs, new approaches emerged for the detection of 2′-*O*-methylation. The observation that RT-enzymes are stalled or paused at a 2′-*O*-Me at low low [deoxynucleotide triphosphate (dNTP)] opened a new way for specific detection [12,43,44]. These techniques were successfully applied to the analysis of various eukaryotic rRNAs [45,46,47] (Figure 3a). More recently, the same approach, although using fluorescently labeled DNA oligonucleotide, was used to complete human rRNA 2′-*O*-methylation mapping [48].

Quantification of low [dNTP] RT stops by quantitative reverse transcription coupled with PCR amplification (qRT-PCR), the so-called RTL-P approach [49], Figure 3b) was proposed for relative quantification of 2′-*O*-methylation site by site. More recently, an engineered KlenTaq RT-enzyme, specific to 2′-*O*-methylation, was developed. It can now replace low [dNTP] conditions, since the mutant enzyme is sensitive to 2′-*O*-methylations and is stalling at those residues even at normal dNTP concentrations [50].

#### 2.2.3. Altered Enzymatic Activity with 2′-*O*-Me RNA 3′-Termini

The presence of a 2′-*O*-Me group may also affect the enzymatic activity at the RNA 3′-termini. First of all, a terminal 2′-*O*-methylation inhibits RNA ligase activity and thus reduces ligation efficiency at the 3′-end [8,9], introducing a considerable ligation bias during sequencing library preparation. This bias is rather annoying for global transcriptome-wide studies, but very useful for analysis of terminal miRNA 2′-*O*-methylation. Interestingly, T4 DNA ligase which can ligate RNAs in a duplex with DNA, is also sensitive to the presence of terminal RNA modifications, in particular 2′-*O*-methylations. Thus this property was used for the development of a ligation-based approach to analyze RNA modifications [39,51]. In addition, 2′-*O*-Me group at the 3′-termini reduces the efficiency of miRNA polyadenylation by polyA-polymerase [10] and this allows direct measurement of miRNA and piRNA 3′-terminal 2′-*O*-methylation [11].

## 3. Limitations of Classical Detection Methods

These traditional methods for RNA 2′-*O*-methylation analysis have numerous limitations. Many of them are rather sensitive, and, in turn, require the use of radioactively labeled RNA, while the others use fluorescent-based detection, but with considerable loss of sensitivity.

- The required amounts of input RNA are quite substantial and a purification step is generally indispensable, making analysis possible only for highly abundant RNAs.
- RT-based methods are relatively sensitive, but generate multiple false-positive as well as false-negative signals [12,44]. Moreover, partial methylation is difficult to detect.
- Quantification is difficult and all approaches are laborious, time consuming and do not allow high-throughput analyses.

## 4. Deep Sequencing-based Approaches

**Figure 4:**
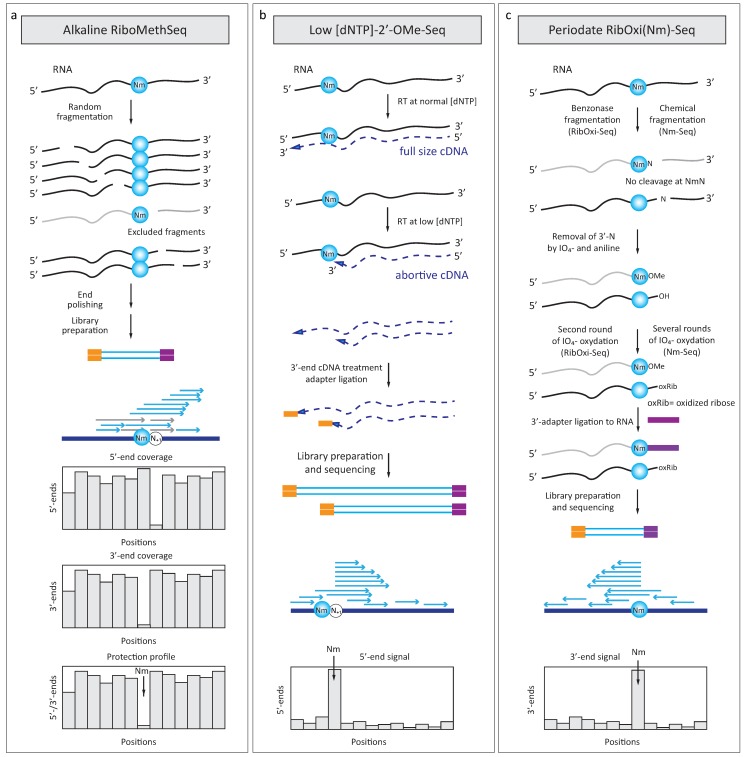
Deep sequencing-based approaches for detection of 2′-*O*-methylated residues in RNA: (**a**) Alkaline fragmentation of RNA used in RiboMethSeq excludes RNA fragments ending with 2′-*O*-Me and, subsequently, also starting with N + 1 nucleotide. After conversion to the sequencing library these fragments become underrepresented (shown in grey). When sequencing reads are mapped to the reference sequence, 5′-end and 3′-end coverage show a characteristic drop, resulting from protection. These profiles are merged together (with -1 nt backshift for the 5′-end coverage) to give a cumulated profile used for calculation of RiboMethSeq scores; (**b**) Detection of 2′-*O*-Me dependent RT stops by 2′-OMe-Seq. Primer extension is done on the same RNA template under normal and reduced [dNTP] and specific low-[dNTP] signals are detected after sequencing and mapping of reads’ 5′-ends; (**c**) Periodate oxidation-based methods for 2′-*O*-Me detection (RibOxi-Seq and Nm-Seq). RNA is fragmented chemically or by nuclease and exposed to periodate oxidation. *Cis*-diols of unmodified ribose are readily oxidized to dialdehyde structures, while 2′-*O*-methylated ribose residues are resistant to treatment. Since enzymatic (or chemical) fragmentation is considerably biased, oxidation/phosphate removal cycles have to be repeated several times to get substantial enrichment of “oxidation-resistant” 3′-ends. Finally, 3′-adapter is directly ligated to the methylated RNA 3′-end, providing the signal after mapping and counting of the sequencing reads (read2 in this case).

In order to improve and accelerate detection and quantification of RNA 2′-*O*-methylation, three deep sequencing-based analytical approaches were proposed; all three exploit different particular properties of 2′-*O*-methylations (Figure 4).

### 4.1. RiboMethSeq

All variants of published RiboMethSeq procedures are based on deep sequencing measurement of 2′-*O*-methylation-induced protection at the 3′-adjacent phosphodiester RNA bond against cleavage at alkaline conditions [52,53,54]. After a random alkaline hydrolysis and optional enrichment of short fragments, RNA pieces are converted to sequencing library using appropriate 3′- and 5′-adapter ligation protocol (Figure 4a). Sequencing is performed either in paired-end or in single read mode and obtained reads are mapped to the reference sequence using precise end-to-end alignment mode, to determine the exact locations of fragments’ 5′- and 3′-ends. A number of these extremities at every position is counted and the resulting (5′/3′- or, sometimes, 5′-only) coverage profile is used to calculate protection (methylation) scores, allowing detection and rather precise quantification of the protection (methylation) level.

Despite general similarity, the exact protocols used for library preparation and bioinformatics analysis pipelines are different in RiboMethSeq versions, probably explaining some minor discrepancies reported. The original RiboMethSeq procedure [52,55] used the proprietary ligation protocol, exploiting ribozymes reactivity and mutant RNA ligase for direct ligation to 5′-OH and 3′-P extremities resulting from alkaline hydrolysis. This allows to avoid minor biases related to subsequent 3′-end de-phosphorylation and 5′-end re-phosphorylation steps used in other protocols; however, the relative inefficiency of the ligation protocol imposes substantial amounts of input RNA (>1 µg, see below).

Optimization of all steps and replacement of direct ligation steps by highly efficient ligation protocol routinely used in small RNA sequencing (e.g., NEB Small RNA kit, New England Biolabs, Ipswich, MA, USA) reduced the amount of required material by almost 1000 fold and greatly simplified the whole analysis pipeline [53].

The currently implemented protocol for RiboMethSeq becomes compatible with low input amounts of RNA, does not use time-consuming and laborious gel-purification steps and provides a reliable quantification of the modification level with only moderate sequencing depth and global analysis cost.

For the moment only RiboMethSeq protocols were extensively applied to profiling and analysis of RNA 2′-*O*-methylation dynamics in rRNA, tRNA and other cellular RNAs (see below Applications).

### 4.2. 2′-OMe-Seq

The 2′-OMe-Seq protocol uses the deep sequencing mapping of RT stops generated by primer extension at low [dNTP] (Figure 4b). Abortive complementary DNA (cDNA) chains obtained under these conditions are converted to sequencing library and the cDNA 3′-ends are determined by mapping to the reference sequence [56]. Comparison with normal RT extension profile at standard [dNTP] allows to exclude some false-positives hits related to RNA structure and sequence.

### 4.3. RibOxi-Seq and Nm-Seq

Two independently published protocols RibOxi-Seq [57] and Nm-Seq [58,59] both exploit the resistance of 2′-*O*-methylated 3′-terminal riboses to periodate cleavage (IO_4_^−^). RNA is first randomly fragmented by a nuclease (benzonase for RibOxi-Seq or fragmentation reagent followed by end repair for Nm-Seq) leaving 5′-phosphates and 3′-OH extremities and these RNA fragments are subjected to periodate oxidation (Figure 4c). Protected 2′-*O*-Me 3′-termini are quite resistant to periodate, but all unmodified *cis*-diol riboses are destroyed and converted to dialdehydes (see Figure 1b). These oxidized riboses are not anymore competent to 3′-adapter ligation and thus, they were excluded from the generated library, allowing enrichment of only 2′-*O*-methylated extremities in the obtained sequencing reads. However, the nuclease or reagent used for fragmentation certainly has preferential recognition sequences and thus RNA cleavage is not really random. In addition, the cleavage exactly at the 2′-*O*-methylated nucleotides is highly inefficient or almost totally absent. To overcome these biases, multiple repetitive cycles of oxydation/β-elimination/de-phosphorylation are required (up to 8 cycles), considerably increasing the loss of material in these treatment steps. Therefore, substantial amount of input RNA is generally required for these applications. In addition, the presence of 2′-*O*-Me at the 3′-extremity is known to reduce the efficiency of 3′-adapter ligation (see above), thus further reducing the library yield and representativity.

## 5. Specific Features of Deep Sequencing Methods

### 5.1. Area of Applications

Even if deep sequencing-based methods for RNA 2′-*O*-methylation analysis were developed only recently, some of these approaches are already extensively used for RNA modification profiling under different physiological conditions. For the moment, the most popular application undoubtedly remains profiling of 2′-*O*-methylations in eukaryotic rRNAs by different versions of RiboMethSeq (Table 1). Since rRNA represents almost 90% of total RNA in almost any cell type, this analysis can be straightforwardly performed directly on total RNA, without preliminary fractionation or enrichment, and at a moderate sequencing depth and cost. Since human ribosome contains at least 110 confirmed 2′-*O*-methylation sites, this paves the way for studies of rRNA 2′-*O*-methylation dynamics under various physiological conditions and in biomedical applications on pathologies. Examples of such modulations have been recently published [60,61,62,63]. Other natural targets for RiboMethSeq are tRNAs from all organisms, since these small non-coding RNAs (ncRNAs) contain a number of known 2′-*O*-methylation sites. Transfer RNA analysis is more complicated than the ones for rRNA, but when positions of modified residues are known, high-throughput quantification of tRNA 2′-*O*-methylation can be reliably performed [64]. It was also demonstrated that with increased sequencing depth even low abundant ncRNAs (like human snRNAs) can be directly accessed in total RNA fraction [65], opening the way for analysis of other low abundant RNA types.

For the moment alternative orthogonal approaches (2′-OMe-Seq, RibOxi-Seq and Nm-Seq) are less widespread, certainly due to excessive amount of input RNA required for analysis (see below), precluding their massive application in biomedical research.

### 5.2. Specificity and Sensitivity of 2′-*O*-Methylation Detection

Direct comparison of these high-throughput methods for the performance in ab initio discovery of modified residues is not easy, since their validation was generally performed with different model RNAs (most used yeast *S. cerevisiae* and human rRNAs) and, in addition, using different subsets of ‘previously validated’ modification sites. Since eukaryotic rRNA may also change its methylation status depending on the cell line used and even upon cultivation conditions and media composition, false negative hits may be also explained by such undermethylation. Moreover, different metrics for performance measurements were used, like Receiver Operating Characteristic (ROC) curve parameters (maximal Matthews Correlation Coefficient, MCC and/or Area Under the Curve, AUC), as well as more simpler threshold level-based models defining validation of sites as candidates.

### 5.3. Required Amount of Input RNA

High-throughput approaches differ very considerably by the required amount of input RNA for analysis. This seems to be a minor issue for many basic research projects, where a substantial amount of input RNA is easily obtained, but strongly limits application of otherwise promising techniques for biomedical projects (e.g., human clinical samples), where biological material is precious and extremely limited.

The best sensitivity was reported so far for variants of RiboMethSeq. Depending on the library preparation protocol this approach requires as low as 10 ng of total human RNA for complete analysis of rRNA 2′-*O*-methylations [53]. Routine analysis is performed with 50 ng, which is quite compatible with many biomedical projects. Other versions of RiboMethSeq are a bit less sensitive, however, they still fit into single digit µg range [54,60]. As anticipated, RT-based 2′-OMe-Seq provides comparable sensitivity, but still requires 2 × 2 µg for the analysis of a single sample [56]. Finally, two versions employing IO_4_^−^-based oxidation require the highest amount of input material, original publications used 7.5 µg (RibOxi-Seq) [57] or 10 µg (Nm-Seq) of total human RNA for rRNA analysis or the same amount of mRNA polyA-selected fraction for a transcriptome-wide study [58,59].

### 5.4. Required Depth of Sequencing

At the first glance, RiboMethSeq analysis requires the highest sequencing coverage since the signal is defined as a protection of a given phosphodiester bond against cleavage, compared to surrounding RNA positions. In principle, average coverage of 5′-/3′-ends of about 100 would be sufficient for reliable analysis, which is about 750,000 reads for human rRNA. However, this reasoning does not take into account irregularity of cleavage due to highly structured rRNA regions. In practice, about 20 times more raw reads are required for a reliable coverage of all rRNA positions. Thus we routinely use the coverage of 12–15 mln of raw reads for the analysis of human rRNA or tRNAs by RiboMethSeq [53,64]. Similar sequencing depth has been used by others [54,60].

Despite the expected enrichment of the signal due to specific detection of RT-stops (2′-OMe-Seq) or protected methylated RNA 3′-end (RibOxi-Seq and Nm-Seq), the reported sequencing depth for analysis appears to be quite similar, ranging from 10–15 mln of raw reads for 2′-OMe-Seq to 15–40 mln of raw reads for RibOxi-Seq and Nm-Seq [57,58].

### 5.5. Quantification of the Methylation Level

Precise quantification of 2′-*O*-methylation level and thus analysis of modification dynamics in RNA is possible only with RiboMethSeq, since the protection signal linearly depends on the methylation level [53,66]. Technical and biological replicates demonstrated that the average standard deviation for yeast or human rRNA 2′-*O*-methylation sites is close to 5% and only very few sites show higher dispersion. In practice >10% of difference of calculated MethScore (ScoreC in previous publications) can be considered as statistically significant. Absolute quantification of methylation level can be achieved if the exact values of MethScore in the absence of modification are known (unmodified transcripts or RNA from knock-out (KO) strains). In vitro transcripts were used for calibration of yeast and human rRNA modification levels, while yeast and *Escherichia coli* strains deleted for the corresponding RNA modification enzymes are useful for tRNA analysis [64,66].

In addition to RiboMethSeq, 2′-OMeSeq can also provide some relative quantification with appropriate spike-in of in vitro RNA transcripts, even if the absolute quantification remains impossible [56]. In contrast, methods based on enrichments of methylated RNA 3′-ends (RibOxi-Seq and Nm-Seq) do not provide any quantitative information.

### 5.6. Sequencing and Bioinformatics Issues

Different technological platforms can be used for sequencing of generated amplicons (libraries). Illumina sequencing (generally HiSeq or NextSeq sequencers) remains the most popular in the field, though PGM/Ion Proton devices are also suitable. Standard RiboMethSeq requires only single-read 50 nt sequencing mode (SR50), paired-end sequencing does not substantially improve the results. Similarly, only single read sequencing mode is in principle required for 2′-OMe-Seq, since only 3′-cDNA ends are of interest (see Figure 4b). In contrast, for RibOxi-Seq and Nm-Seq paired end sequencing is mandatory, since important information resides in the beginning of the read2 in paired-end mode.

Data treatment and analysis steps are similar in all approaches, reads’ processing generally begins with trimming, followed by alignment to the reference sequence and counting of 5′- or 3′- (or both) ends of mapped reads. A special care should also be taken at the mapping step to avoid multiply mapped sequences of unknown origin.

## 6. Conclusions

Combination of traditional and deep sequencing-based approaches for RNA 2′-*O*-methylation analysis now opens the way for an exhaustive identification of novel modified sites in diverse cellular RNAs as well as careful investigations of RNA 2′-*O*-methylation dynamics under various physiological conditions and in human pathologies related to RNA modifications.

## Figures and Tables

**Table 1:** Comparison of published deep sequencing–based methods for 2′-*O*-Me detection.

	RiboMethSeq [53]	2′-OMe-Seq [56]	RibOxi-Seq [57]	(Nm-Seq) [58,59]
Described applications	rRNA, tRNA, (snRNA)	rRNA	rRNA	rRNA, mRNA
RNA input	10 ng (50 ng in routine)	2 × 2 µg	7.5 µg	10 µg rRNA 10 µg polyA mRNA
Sequencing depth	~1000 reads/RNA position (10–15 mln raw reads/sample)	10–15 mln reads/sample	10–15 mln reads/sample	10–15 mln reads/sample
Quantification	Yes	Yes (relative only)	No	No
Sequencing mode used	Single read 50 nt		Paired end 2 × 75 nt	

transfer RNAs (tRNAs), ribosomal RNAs (rRNAs), small nuclear RNAs (snRNAs), messenger RNAs (mRNAs).

## Supplementary Materials

The following are available online at http://www.mdpi.com/2073-4425/9/12/642/s1, Figure S1: Comparative analysis of the 2′-*O*-methylation levels obtained by mass-spectrometry analysis and MethScore for the same position provided by various RiboMethSeq protocols.

## References

[1] Ayadi L., Galvanin A., Pichot F., Marchand V., Motorin Y. (2019). RNA ribose methylation (2′-*O*-methylation): Occurrence, biosynthesis and biological functions. BBA Gene Regul. Mech..

[2] Kawai G., Ue H., Yasuda M., Sakamoto K., Hashizume T., McCloskey J.A., Miyazawa T., Yokoyama S. (1991). Relation between functions and conformational characteristics of modified nucleosides found in tRNAs. Nucl. Acids Symp. Ser..

[3] Kawai G., Yamamoto Y., Kamimura T., Masegi T., Sekine M., Hata T., Iimori T., Watanabe T., Miyazawa T., Yokoyama S. (1992). Conformational rigidity of specific pyrimidine residues in tRNA arises from posttranscriptional modifications that enhance steric interaction between the base and the 2′-hydroxyl group. Biochemistry.

[4] Prusiner P., Yathindra N., Sundaralingam M. (1974). Effect of ribose O(2′)-methylation on the conformation of nucleosides and nucleotides. Biochim. Biophys. Acta.

[5] Monaco P.L., Marcel V., Diaz J.-J., Catez F. (2018). 2′-*O*-Methylation of Ribosomal ribosomal RNA: Towards an epitranscriptomic control of translation?. Biomolecules.

[6] Natchiar S.K., Myasnikov A.G., Hazemann I., Klaholz B.P. (2018). Visualizing the role of 2′-OH rRNA methylations in the human ribosome structure. Biomolecules.

[7] Yu B., Chen X. (2010). Analysis of miRNA Modifications. Methods Mol. Biol..

[8] Munafó D.B., Robb G.B. (2010). Optimization of enzymatic reaction conditions for generating representative pools of cDNA from small RNA. RNA.

[9] Dard-Dascot C., Naquin D., d’Aubenton-Carafa Y., Alix K., Thermes C., van Dijk E. (2018). Systematic comparison of small RNA library preparation protocols for next-generation sequencing. BMC Genom..

[10] Yang Z., Ebright Y.W., Yu B., Chen X. (2006). HEN1 recognizes 21–24 nt small RNA duplexes and deposits a methyl group onto the 2′ OH of the 3′ terminal nucleotide. Nucl. Acids Res..

[11] Wang N., Qu S., Sun W., Zeng Z., Liang H., Zhang C.-Y., Chen X., Zen K. (2018). Direct quantification of 3′ terminal 2′-*O*-methylation of small RNAs by RT-qPCR. RNA.

[12] Maden B.E. (2001). Mapping 2′-*O*-methyl groups in ribosomal RNA. Methods.

[13] Baskin F., Dekker C.A. (1967). A rapid and specific assay for sugar methylation in ribonucleic acid. J. Biol. Chem..

[14] Trim A.R., Parker J.E. (1972). Nucleotide sequence in fourteen dinucleotides, modified by 2′-*O*-methylation, from yeast ribonucleic acid, determined by periodate degradation and by pentose analysis. Anal. Biochem..

[15] Abbate J., Rottman F. (1972). Gas chromatographic method for determination of 2′-*O*-methylation in RNA. Anal. Biochem..

[16] Sardana M.K., Fuke M. (1980). A rapid procedure to determine the content of 2′-*O*-methylation in RNA by homochromatography. Anal. Biochem..

[17] Qiu F., McCloskey J.A. (1999). Selective detection of ribose-methylated nucleotides in RNA by a mass spectrometry-based method. Nucl. Acids Res..

[18] Kirpekar F., Hansen L.H., Rasmussen A., Poehlsgaard J., Vester B. (2005). The archaeon *Haloarcula marismortui* has few modifications in the central parts of its 23S ribosomal RNA. J. Mol. Biol..

[19] Takeda N., Nakamura M., Yoshizumi H., Tatematsu A. (1994). Detection of ribose-methylated nucleotides in *Pyrodictium occultum* tRNA by liquid chromatography—frit-fast atom bombardment mass spectrometry. J. Chromatogr. B Biomed. Sci. Appl..

[20] Zhang Q., Wang Y. (2006). Differentiation of 2′-*O*- and 3′-*O*-methylated ribonucleosides by tandem mass spectrometry. J. Am. Soc. Mass. Spectrom..

[21] Kellner S., Burhenne J., Helm M. (2010). Detection of RNA modifications. RNA Biol..

[22] Gaston K.W., Limbach P.A. (2014). The identification and characterization of non-coding and coding RNAs and their modified nucleosides by mass spectrometry. RNA Biol..

[23] Jora M., Lobue P.A., Ross R.L., Williams B., Addepalli B. (2018). Detection of ribonucleoside modifications by liquid chromatography coupled with mass spectrometry. Biochim. Biophys. Acta Gene Regul. Mech..

[24] Cavaillé J., Chetouani F., Bachellerie J.P. (1999). The yeast *Saccharomyces cerevisiae* YDL112w ORF encodes the putative 2′-*O*-ribose methyltransferase catalyzing the formation of Gm18 in tRNAs. RNA.

[25] Bonnerot C., Pintard L., Lutfalla G. (2003). Functional redundancy of Spb1p and a snR52-dependent mechanism for the 2′-*O*-ribose methylation of a conserved rRNA position in yeast. Mol. Cell.

[26] Pintard L., Lecointe F., Bujnicki J.M., Bonnerot C., Grosjean H., Lapeyre B. (2002). Trm7p catalyses the formation of two 2′-*O*-methylriboses in yeast tRNA anticodon loop. EMBO J..

[27] Brand R.C., Klootwijk J., Van Steenbergen T.J., De Kok A.J., Planta R.J. (1977). Secondary methylation of yeast ribosomal precursor RNA. Eur. J. Biochem..

[28] Maden B.E. (1986). Identification of the locations of the methyl groups in 18 S ribosomal RNA from *Xenopus laevis* and man. J. Mol. Biol..

[29] Maden B.E. (1988). Locations of methyl groups in 28S rRNA of *Xenopus laevis* and man. Clustering in the conserved core of molecule. J. Mol. Biol..

[30] Yang J., Sharma S., Kötter P., Entian K.-D. (2015). Identification of a new ribose methylation in the 18S rRNA of *S. cerevisiae*. Nucl. Acids Res..

[31] Yang J., Sharma S., Watzinger P., Hartmann J.D., Kötter P., Entian K.-D. (2016). Mapping of complete set of ribose and base modifications of yeast rRNA by RP-HPLC and mung bean nuclease assay. PLoS ONE.

[32] Taoka M., Nobe Y., Yamaki Y., Yamauchi Y., Ishikawa H., Takahashi N., Nakayama H., Isobe T. (2016). The complete chemical structure of *Saccharomyces cerevisiae* rRNA: Partial pseudouridylation of U2345 in 25S rRNA by snoRNA snR9. Nucl. Acids Res..

[33] Taoka M., Nobe Y., Yamaki Y., Sato K., Ishikawa H., Izumikawa K., Yamauchi Y., Hirota K., Nakayama H., Takahashi N. (2018). Landscape of the complete RNA chemical modifications in the human 80S ribosome. Nucl. Acids Res..

[34] Singh H., Lane B.G. (1964). The alkali-stable dinucleotide sequences in 18S + 28S ribonucleates from wheat germ. Can. J. Biochem..

[35] Trim A.R., Parker J.E. (1970). Preparation, purification and analyses of thirteen alkali-stable dinucleotides from yeast ribonucleic acid. Biochem. J..

[36] Tycowski K.T., Smith C.M., Shu M.D., Steitz J.A. (1996). A small nucleolar RNA requirement for site-specific ribose methylation of rRNA in *Xenopus*. Proc. Natl. Acad. Sci. USA.

[37] Kiss-László Z., Henry Y., Bachellerie J.P., Caizergues-Ferrer M., Kiss T. (1996). Site-specific ribose methylation of preribosomal RNA: A novel function for small nucleolar RNAs. Cell.

[38] Motorin Y., Muller S., Behm-Ansmant I., Branlant C. (2007). Identification of modified residues in RNAs by reverse transcription-based methods. Meth. Enzymol..

[39] Huang C., Karijolich J., Yu Y.-T. (2016). Detection and quantification of RNA 2′-*O*-methylation and pseudouridylation. Methods.

[40] Yu Y.T., Shu M.D., Steitz J.A. (1997). A new method for detecting sites of 2′-*O*-methylation in RNA molecules. RNA.

[41] Lapham J., Yu Y.T., Shu M.D., Steitz J.A., Crothers D.M. (1997). The position of site-directed cleavage of RNA using RNase H and 2′-*O*-methyl oligonucleotides is dependent on the enzyme source. RNA.

[42] Buchhaupt M., Peifer C., Entian K.-D. (2007). Analysis of 2′-*O*-methylated nucleosides and pseudouridines in ribosomal RNAs using DNAzymes. Anal. Biochem..

[43] Lowe T.M., Eddy S.R. (1999). A computational screen for methylation guide snoRNAs in yeast. Science.

[44] Maden B.E., Corbett M.E., Heeney P.A., Pugh K., Ajuh P.M. (1995). Classical and novel approaches to the detection and localization of the numerous modified nucleotides in eukaryotic ribosomal RNA. Biochimie.

[45] Rebane A., Roomere H., Metspalu A. (2002). Locations of several novel 2′-*O*-methylated nucleotides in human 28S rRNA. BMC Mol. Biol..

[46] Higa S., Maeda N., Kenmochi N., Tanaka T. (2002). Location of 2(‘)-*O*-methyl nucleotides in 26S rRNA and methylation guide snoRNAs in *Caenorhabditis elegans*. Biochem. Biophys. Res. Commun..

[47] Piekna-Przybylska D., Decatur W.A., Fournier M.J. (2008). The 3D rRNA modification maps database: With interactive tools for ribosome analysis. Nucl. Acids Res..

[48] Filippova J.A., Stepanov G.A., Semenov D.V., Koval O.A., Kuligina E.V., Rabinov I.V., Richter V.A. (2015). Modified method of rRNA structure analysis reveals novel characteristics of box C/D RNA analogues. Acta Nat..

[49] Dong Z.-W., Shao P., Diao L.-T., Zhou H., Yu C.-H., Qu L.-H. (2012). RTL-P: A sensitive approach for detecting sites of 2′-*O*-methylation in RNA molecules. Nucl. Acids Res..

[50] Aschenbrenner J., Marx A. (2016). Direct and site-specific quantification of RNA 2′-*O*-methylation by PCR with an engineered DNA polymerase. Nucl. Acids Res..

[51] Saikia M., Dai Q., Decatur W.A., Fournier M.J., Piccirilli J.A., Pan T. (2006). A systematic, ligation-based approach to study RNA modifications. RNA.

[52] Birkedal U., Christensen-Dalsgaard M., Krogh N., Sabarinathan R., Gorodkin J., Nielsen H. (2015). Profiling of ribose methylations in RNA by high-throughput sequencing. Angew. Chem. Int. Ed..

[53] Marchand V., Blanloeil-Oillo F., Helm M., Motorin Y. (2016). Illumina-based RiboMethSeq approach for mapping of 2′-*O*-Me residues in RNA. Nucl. Acids Res..

[54] Gumienny R., Jedlinski D.J., Schmidt A., Gypas F., Martin G., Vina-Vilaseca A., Zavolan M. (2017). High-throughput identification of C/D box snoRNA targets with CLIP and RiboMeth-seq. Nucl. Acids Res..

[55] Krogh N., Birkedal U., Nielsen H. (2017). RiboMeth-seq: Profiling of 2′-*O*-Me in RNA. Methods Mol. Biol..

[56] Incarnato D., Anselmi F., Morandi E., Neri F., Maldotti M., Rapelli S., Parlato C., Basile G., Oliviero S. (2017). High-throughput single-base resolution mapping of RNA 2′-*O*-methylated residues. Nucl. Acids Res..

[57] Zhu Y., Pirnie S.P., Carmichael G.G. (2017). High-throughput and site-specific identification of 2′-*O*-methylation sites using ribose oxidation sequencing (RibOxi-seq). RNA.

[58] Dai Q., Moshitch-Moshkovitz S., Han D., Kol N., Amariglio N., Rechavi G., Dominissini D., He C. (2017). Nm-seq maps 2′-*O*-methylation sites in human mRNA with base precision. Nat. Methods.

[59] Hsu P.J., Fei Q., Dai Q., Shi H., Dominissini D., Ma L., He C. (2018). Single base resolution mapping of 2′-*O*-methylation sites in human mRNA and in 3′ terminal ends of small RNAs. Methods.

[60] Krogh N., Jansson M.D., Häfner S.J., Tehler D., Birkedal U., Christensen-Dalsgaard M., Lund A.H., Nielsen H. (2016). Profiling of 2′-*O*-Me in human rRNA reveals a subset of fractionally modified positions and provides evidence for ribosome heterogeneity. Nucl. Acids Res..

[61] Erales J., Marchand V., Panthu B., Gillot S., Belin S., Ghayad S.E., Garcia M., Laforêts F., Marcel V., Baudin-Baillieu A. (2017). Evidence for rRNA 2′-*O*-methylation plasticity: Control of intrinsic translational capabilities of human ribosomes. Proc. Natl. Acad. Sci. USA.

[62] Zhou F., Liu Y., Rohde C., Pauli C., Gerloff D., Köhn M., Misiak D., Bäumer N., Cui C., Göllner S. (2017). AML1-ETO requires enhanced C/D box snoRNA/RNP formation to induce self-renewal and leukaemia. Nat. Cell Biol..

[63] Sharma S., Marchand V., Motorin Y., Lafontaine D.L.J. (2017). Identification of sites of 2′-*O*-methylation vulnerability in human ribosomal RNAs by systematic mapping. Sci. Rep..

[64] Marchand V., Pichot F., Thüring K., Ayadi L., Freund I., Dalpke A., Helm M., Motorin Y. (2017). Next-Generation Sequencing-Based RiboMethSeq Protocol for Analysis of tRNA 2′-*O*-Methylation. Biomolecules.

[65] Krogh N., Kongsbak-Wismann M., Geisler C., Nielsen H. (2017). Substoichiometric ribose methylations in spliceosomal snRNAs. Org. Biomol. Chem..

[66] Ayadi L., Motorin Y., Marchand V. (2018). Quantification of 2′-*O*-Me residues in RNA using next-generation sequencing (Illumina RiboMethSeq Protocol). Methods Mol. Biol..

