# Supplementary material for: Detection and Analysis of RNA Ribose 2′-O-Methylations: Challenges and Solutions
Source: Genes (Basel). 2018 Dec 18;9(12):642. doi: 10.3390/genes9120642 (PMC6316082; doi:10.3390/genes9120642)
Supplement: Supplementary file 1 [file genes-09-00642-s001.pdf]

Supplementary information

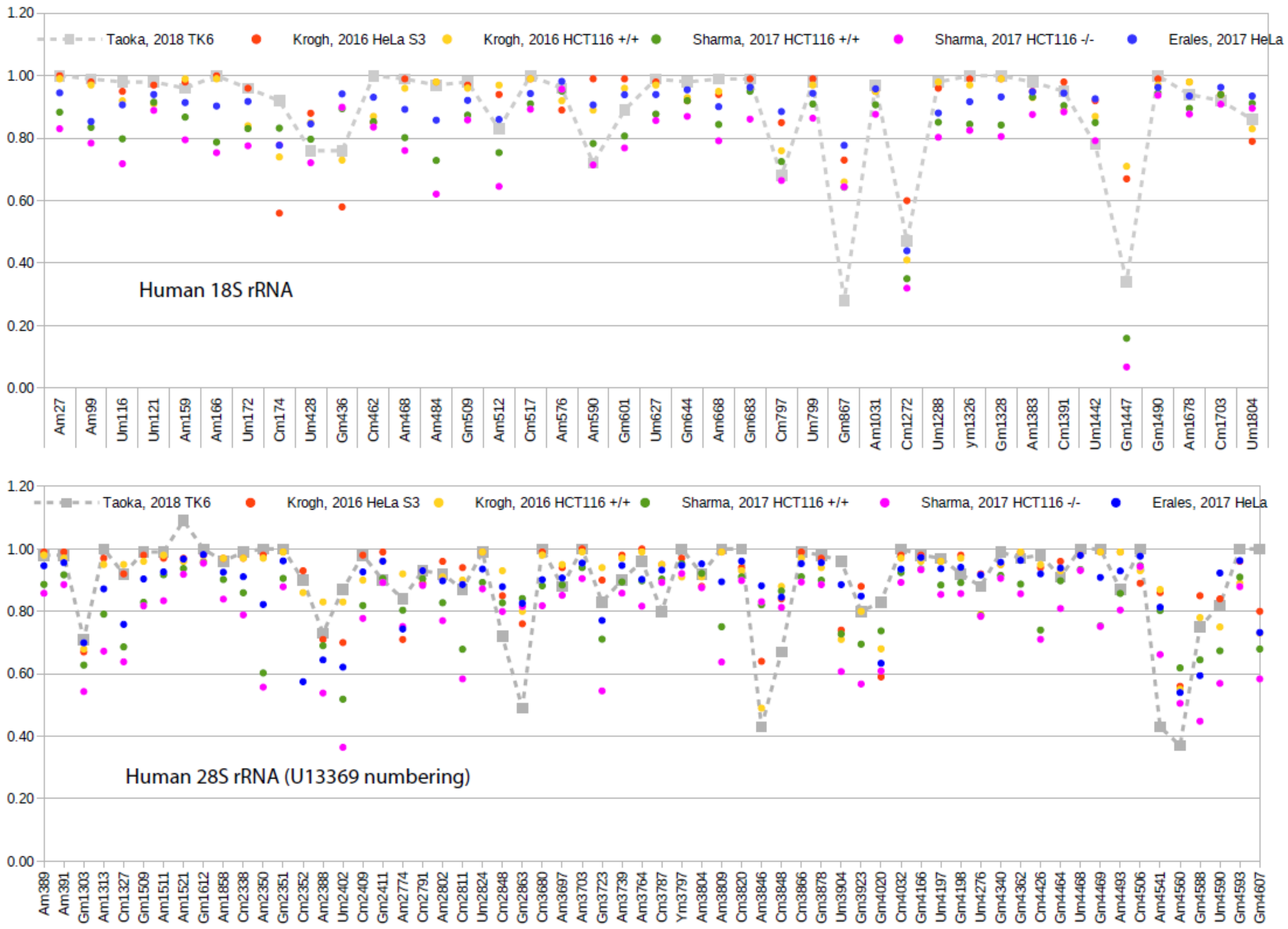

Figure S1. Comparative analysis of the 2′-O-methylation levels obtained by mass-spectrometry analysis and MethScore for the same position provided by various RiboMethSeq protocols. Direct comparison seems to be difficult since different cell lines were used and culture conditions are not necessarily identical. Cell lines used:

TK6 human lymphoblast cell from spleen, Epstein-Barr virus transformed [1], cervix adenocarcinoma cell HeLa S3 (ATCC CCL-2.2) and colorectal carcinoma cell line HCT116 (ATCC CCL-247) [2], colorectal carcinoma cell line HCT116 (ATCC CCL-247) (HCT +/+) and isogenous cell line in which endogenous alleles of p53 were disrupted sequentially by homologous recombination (HCT -/-) [3], cervix adenocarcinoma cell line HeLa [4].

1. Taoka, M.; Nobe, Y.; Yamaki, Y.; Sato, K.; Ishikawa, H.; Izumikawa, K.; Yamauchi, Y.; Hirota, K.; Nakayama, H.; Takahashi, N.; et al. Landscape of the complete RNA chemical modifications in the human 80S ribosome. *Nucleic Acids Res.* **2018**, *46*, 9289–9298.
2. Krogh, N.; Jansson, M.D.; Häfner, S.J.; Tehler, D.; Birkedal, U.; Christensen-Dalsgaard, M.; Lund, A.H.; Nielsen, H. Profiling of 2′-O-Me in human rRNA reveals a subset of fractionally modified positions and provides evidence for ribosome heterogeneity. *Nucleic Acids Res.* **2016**, *44*, 7884–7895.
3. Sharma, S.; Marchand, V.; Motorin, Y.; Lafontaine, D.L.J. Identification of sites of 2′-O-methylation vulnerability in human ribosomal RNAs by systematic mapping. *Sci Rep* **2017**, *7*, 11490.
4. Erales, J.; Marchand, V.; Panthu, B.; Gillot, S.; Belin, S.; Ghayad, S.E.; Garcia, M.; Laforêts, F.; Marcel, V.; Baudin-Baillieu, A.; et al. Evidence for rRNA 2′-O-methylation plasticity: Control of intrinsic translational capabilities of human ribosomes. *Proc. Natl. Acad. Sci. U.S.A.* **2017**, *114*, 12934–12939.
